# In Vivo Imaging of Lymphatic Drainage of Cerebrospinal Fluid in Mouse

**DOI:** 10.1186/2045-8118-10-35

**Published:** 2013-12-21

**Authors:** Emily Mathieu, Neeru Gupta, R Loch Macdonald, Jinglu Ai, Yeni H Yücel

**Affiliations:** 1 Keenan Research Centre for Biomedical Science, St. Michael’s Hospital, Toronto, ON, Canada; 2 Departments of Laboratory Medicine & Pathobiology and Ophthalmology & Vision Sciences, St. Michael’s Hospital, University of Toronto, Toronto, ON, Canada; 3 Division of Neurosurgery, Department of Surgery, St. Michael’s Hospital, University of Toronto, Toronto, ON, Canada; 4 Labatt Family Centre of Excellence in Brain Injury and Trauma Research, St. Michael’s Hospital, Toronto, ON, Canada

**Keywords:** Cerebrospinal fluid, Lymphatic, Nanotechnology, In vivo imaging, Mouse, Hyperspectral imaging, Non-invasive imaging, Quantum dot

## Abstract

**Background:**

Mouse models are commonly used to study central nervous system disorders, in which cerebrospinal fluid (CSF) drainage may be disturbed. However, mouse CSF drainage into lymphatics has not been thoroughly characterized. We aimed to image this using an *in vivo* approach that combined quantum dot fluorescent nanoparticles with hyperspectral imaging.

**Findings:**

Quantum dot 655 was injected into the CSF of the cisterna magna in seven mice and visualized by *in vivo* hyperspectral imaging at time points 20 and 40 min, 1, 2, and 6 h after injection. In controls (n = 4), quantum dots were applied directly onto intact dura mater covering the cisterna magna. After imaging, lymph nodes in the neck were harvested and processed post-mortem for histological analysis. After injection into the CSF, quantum dot signal was detected *in vivo* in submandibular lymph nodes of all mice studied as early as 20 min, but not in controls. Post-mortem gross and histological examination of lymph nodes confirmed *in vivo* observations.

**Conclusions:**

Non-invasive *in vivo* hyperspectral imaging is a useful tool to study CSF lymphatic drainage and is relevant to understanding this pathway in CNS disease models.

## Findings

### Introduction

Cerebrospinal fluid (CSF) bathes the central nervous system (CNS) and plays roles in a number of important functions, including hydraulic cushioning, nutrient delivery, drainage of waste, and immune surveillance [1]. Altered CSF circulation, mainly due to blockage of CSF outflow, is implicated in hydrocephalus and increased intracranial pressure (ICP) associated with ischemic or hemorrhagic cerebrovascular disease [2]. Tracer studies have shown that CSF drains via arachnoid granulations into the bloodstream and along olfactory nerves into nasal lymphatics [3,4]. Studies of CSF drainage have been performed in cat, rabbit, and sheep due to their relatively large lymphatic vessels that facilitate cannulation and collection experiments [5,6]. Non-invasive imaging modalities such as scintigraphy [7] and computed tomographic scanning [8] have been used to visualize lymphatic drainage of CSF in animals such as cats and rabbits, avoiding the need for surgical exposure of the lymphatic pathway for cannulation or visualization. However, the low optical resolution of such methods has precluded their use to study CSF drainage pathways in mice. Although monitoring drainage of dyes injected into the CSF by direct visualization of surgically exposed cervical lymph nodes has been described [9], the lack of non-invasive *in vivo* methods to assess lymphatic drainage of CSF has hampered our understanding of this pathway in mouse, an important laboratory species for studies of CNS disorders. Optical hyperspectral imaging is a non-invasive, whole animal imaging technique based on the spectral analysis of light reflected by the tissue components. Spectral “unmixing” allows the separation of the tracer signal from the autofluorescence of skin and other tissues. Non-invasive techniques combining hyperspectral imaging with quantum dots (QD) have recently been used to assess lymphatic drainage from the skin [10]. We have used a similar technique to study lymphatic drainage from the eye [11,12]. Here, we investigate the lymphatic drainage of CSF in mice using QD fluorescent nanoparticles injected into the cisterna magna and visualized by hyperspectral imaging *in vivo*.

## Materials and methods

Using a protocol approved by the institutional Animal Care Committee, male 129SVE mice (age 6–10 weeks; n = 11) were shaved (head and neck) and mounted under general anesthesia (1.5% isoflurane inhalation in O_2_) onto a stereotaxic frame (Kopf Instruments, CA, USA) in a prone position and a 1 cm skin incision was made from the base of the neck to the tip of the occiput. Muscles of the neck were bluntly dissected with forceps to expose the dura mater covering the cisterna magna and the incision was held open with retractors. Quantum dot 655 (Qdot® ITK^TM^ Carboxyl Quantum Dots, Invitrogen, OR, USA), fluorescent nanoparticles with a hydrodynamic size of 19 nm, ellipsoid shape, CdSe/ZnS core/shell, and an emission peak at 655 nm, were used as formulated by the manufacturer (8 μM QD in a 50 mM borate buffer). Negatively-charged QDs were chosen because a negative surface charge has been shown to improve lymphatic uptake and retention [13]. In seven mice, using a 25 μL syringe (Hamilton Co., NV, USA) with 33 g needle (20° beveled point, Hamilton Co., NV, USA) mounted on a stereotaxic arm, 3 μL of QD solution was manually injected over 1 min into the CSF of the cisterna magna. Any leaked CSF (approx. 1–2 μL) was absorbed with micro-sponges and the injection site was immediately sealed with cyanoacrylate tissue glue (Vetbond, 3M Inc., MN, USA) to prevent further leakage. Experiments were performed in four additional mice to control for possible drainage of leaked tracer along lymphatics in the posterior cervical tissues. For these control mice, 3 μL of QD was applied onto the intact dura mater surface overlying the cisterna magna. For all mice the skin incision was closed and subcutaneous analgesia with buprenorphine (Temgesic, 0.3 mg/mL, Reckitt Benckiser, Berkshire, UK) 0.1 mg/kg plus 1.0 mL saline for rehydration were given. After the injection, mice were removed from the stereotaxic frame and returned to cages prior to imaging.

In four additional mice, ICP was continuously measured under anesthesia (same as above) before, during, and 10 min after injection of 3 μL of QD into CSF of the cisterna magna, followed by immediate sacrifice by CO_2_ inhalation. A blunt tip needle was connected to a pressure probe and monitoring device (mo. MPM-1, Integra, NJ, USA) through a saline-filled tube and placed above the dura mater in a 1 mm burr hole in the parietal bone and sealed with bone wax. ICP measurements before and after tracer injection were compared using a paired t-test and no significant change was noted when comparing pre-injection to 10 min post-injection ICP, respectively (7.3 ± 0.96 mmHg [Mean ± S.D.] vs. 8.6 ± 1.1 mmHg; n = 4, p = 0.11).

To detect drainage into cervical lymph nodes *in vivo,* mice were imaged under isoflurane anesthesia in the supine position using the protocol described in Tam *et al.* 2011 [11]. Anesthesia was discontinued between imaging sessions, and mice were returned to cages. *In vivo* hyperspectral imaging was performed (Maestro^TM^,CRi, MA, USA) 20 and 40 min, 1, 2 and 6 hours after tracer injection, and mice were sacrificed by CO_2_ inhalation following the final imaging session. Hyperspectral images were analyzed using Maestro 2.4 imaging software unmixing algorithms to visualize QD signal, with creation of a spectral library from emission spectra of QD, tissue autofluorescence, and food autofluorescence.

To confirm the presence and further localize QD in neck tissues, hyperspectral imaging of post-mortem experimental and control mice was performed. After imaging *in vivo*, animals were immersion fixed in 4% paraformaldehyde and cryoprotected in glycerol solution using the protocol from Tam *et al.* 2011 [11]. The head, neck, and upper torso were scanned with skin removed to expose the submandibular salivary glands and associated lymph nodes. All neck tissue with QD signal was harvested and the remainder of the mouse, along with the dissected tissue block, was rescanned. If rescanning revealed signal in the deep neck tissue, the remainder of the neck was harvested. Neck tissue blocks were embedded in cryomatrix and frozen with solid CO_2_ (dry ice). Frozen tissue blocks were serially sectioned (140 μm thick) using a sliding microtome (Leica SM2400, Leica, Germany). Sections were collected on strips of plastic wrap and mounted on charged slides using PVA-DABCO anti-fade mounting medium (polyvinyl alcohol, Sigma-Aldrich, MO, USA; DABCO, MP Biomedicals, CA, USA). All sections were scanned hyperspectrally with reduced exposure time of 300 ms to screen for regions with QD signal.

To validate *in vivo* detection of QD within lymph nodes, immunofluorescence staining for collagen-IV and nuclear counterstaining was performed on neck sections with QD signal using the staining protocol from Tam *et al.* 2013 [12]. Neck sections were analyzed by confocal laser-scanning microscope (TCS SL, Leica, Germany).

Identification of lymph nodes was undertaken using the nomenclature proposed by Van den Broeck and coworkers [14], with the exception that we have used the commonly accepted term of “submandibular lymph nodes” instead of “mandibular lymph nodes”.

## Results

**Figure 1:**
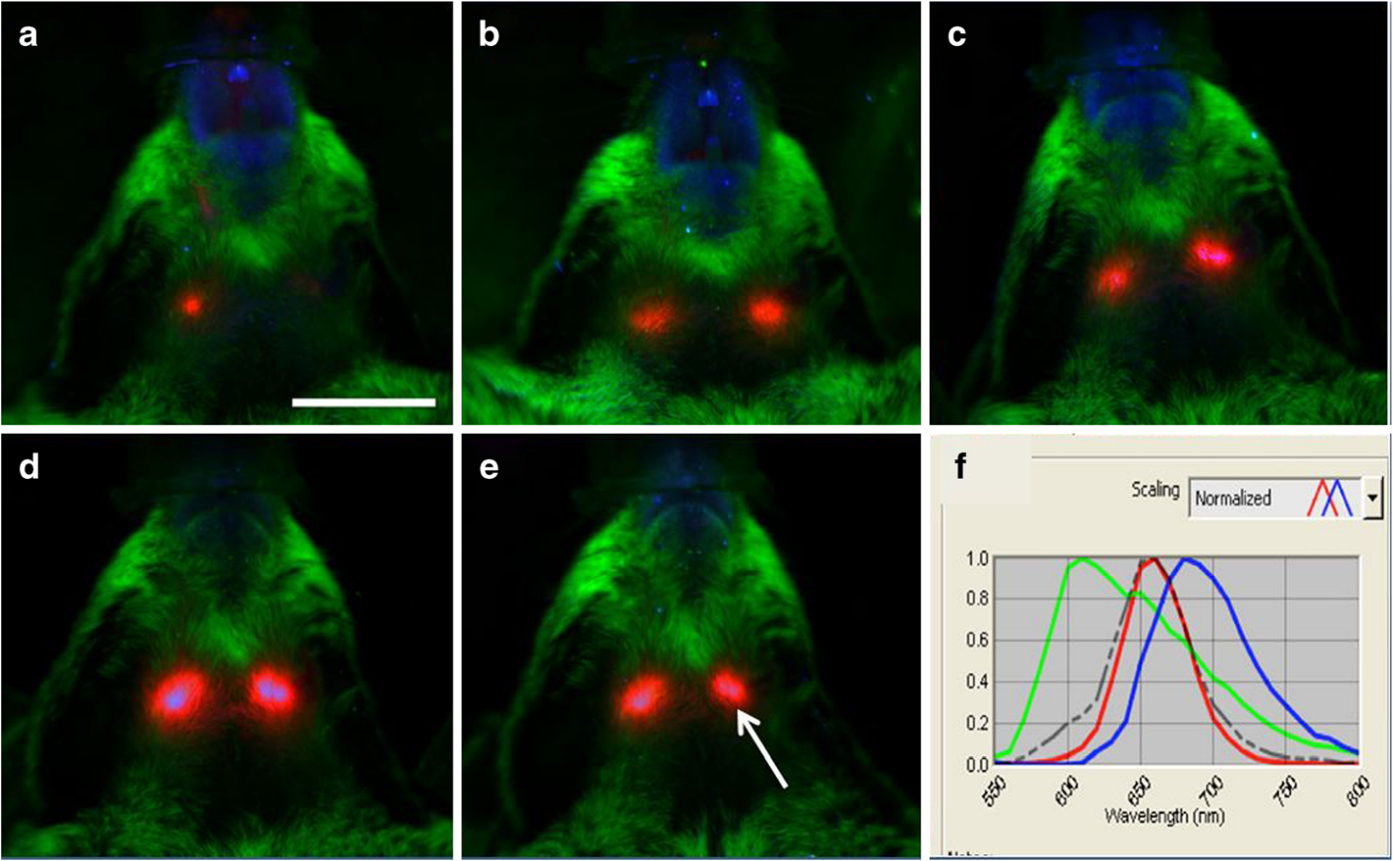
***In vivo *hyperspectral fluorescence images after injection of QD into CSF.** Ventral view of head and neck 20 min **(a)**, 40 min **(b)**, 1 h **(c)**, 2 h **(d)** and 6 h **(e)** following injection of QD into CSF of cisterna magna. QD signal (red) is seen in the submandibular region of the right and left side of the neck. Skin/fur autofluoresence (green), autofluorescence from food (blue). **(f)** Spectral emission profile (grey dotted line) of the region indicated by the arrow in **(e)** shows that the signal matches the profile for QD (red line) and is distinct from that for skin/fur autofluorescence (green line) and that of autofluorescence from food (blue line). Scale = 1 cm.

**Figure 2:**
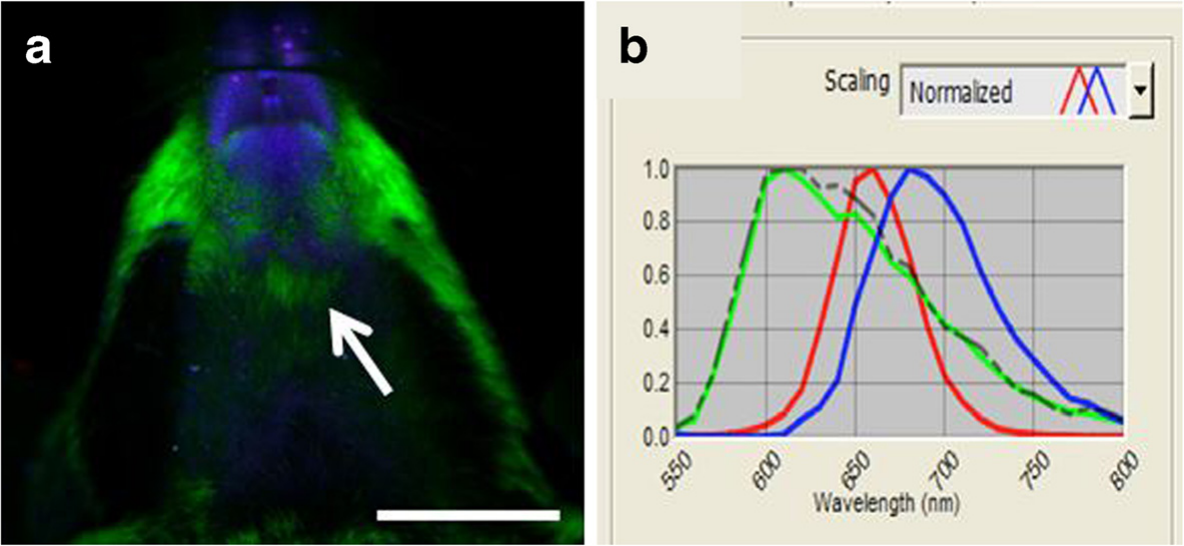
***In vivo *hyperspectral fluorescence image of control mouse. (a)** Ventral view of the head and neck 6 h after QD application onto the dura mater covering the cisterna magna. No QD signal (red) is visible. Skin/fur autofluoresence (green), autofluorescence from food (blue). **(b)** Spectral emission profile (grey dotted line) of the region indicated by the arrow in **(a)** shows that signal in the submandibular region matched the profile for skin/fur autofluorescence (green line) and is distinct from QD signal (red line) and that of autofluorescence from food (blue line). Scale = 1 cm.

With *in vivo* hyperspectral imaging, QD signal was evident in the submandibular region of the right and left sides of the neck by 20 min in 5 of 7 mice and in all mice by 40 min. QD signal remained in the same regions at 1, 2 and 6 h after tracer injection, with peak intensity seen at 1–2 h and a slight decrease by 6 h (Figure 1a-e). The signal was confirmed to match the emission profile of QD by spectral analysis through unmixing algorithms (Figure 1f). QD signal was not detected *in vivo* in the neck region of control mice by 6 h after QD was applied to dura mater covering the cisterna magna (Figure 2a). Spectral analysis of the submandibular region in these mice showed an emission profile matching tissue autofluorescence (Figure 2b).

**Figure 3:**
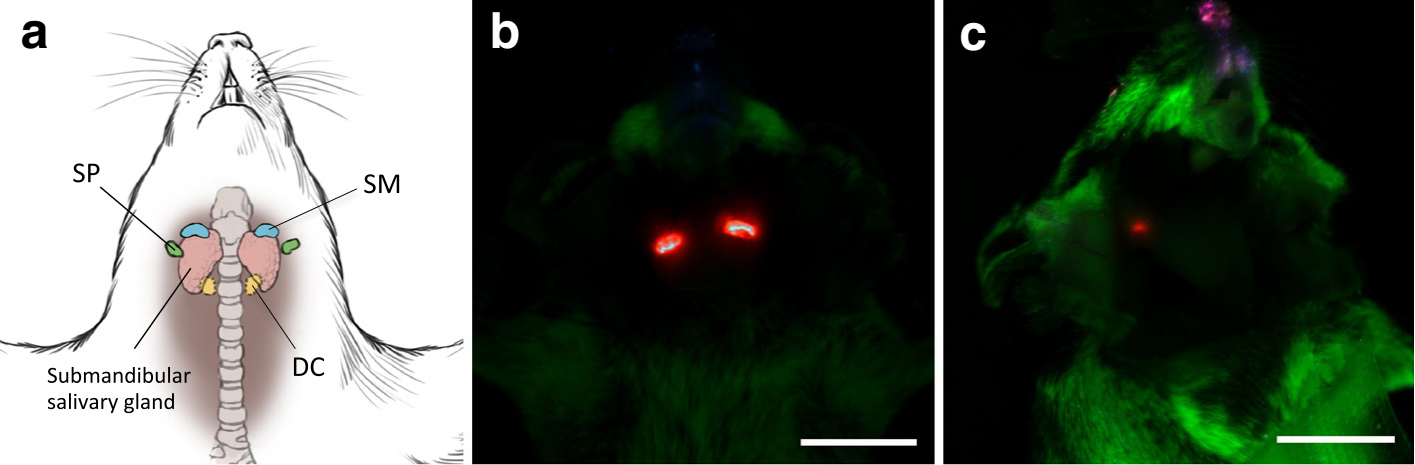
**Post-mortem hyperspectral fluorescence imaging with skin removed. (a)** Diagram of murine lymph nodes in the neck showing locations for the submandibular (SM; blue), superficial parotid (SP; green), and deep cervical (DC; yellow) lymph nodes. **(b)** Ventral view of the upper body and head showing intense QD signal (red) in left and right submandibular region 6 h after QD injection into CSF. **(c)** Right latero-ventral view of the head and neck of a control mouse showing weak QD signal (red) in the right superficial parotid lymph node 6 h after QD application onto the dura mater overlying the cisterna magna. Tissue autofluorescence is shown in green. Scale = 1 cm.

Post-mortem imaging revealed QD in the region of submandibular lymph nodes in all mice 6 hours after CSF injection (Figure 3a,b). After removal of the salivary glands and associated lymph nodes, rescanning of mice revealed focal points of QD signal in the region of the deep cervical lymph nodes in 2 of 7 mice with CSF injections, although drainage to these nodes was not apparent *in vivo.* In control mice with extra-dural QD application, signal was detected post-mortem in the superficial parotid lymph nodes (n = 3/4; Figure 3c) and deep cervical lymph nodes (n = 3/4), but not in submandibular lymph nodes.

**Figure 4:**
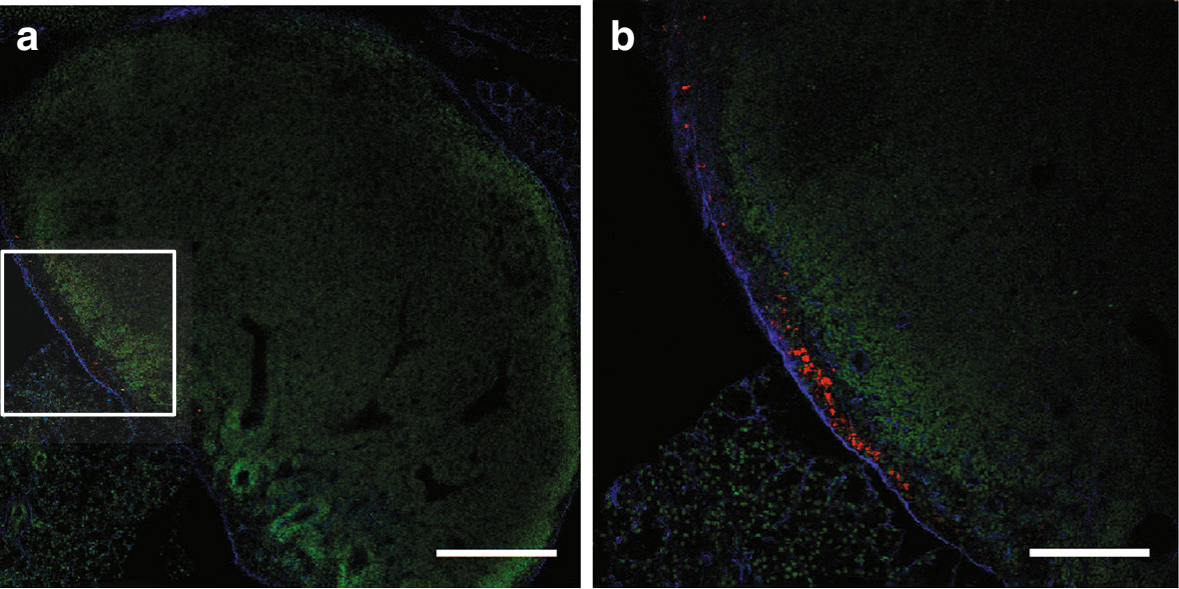
**QD in submandibular lymph nodes 6 hours after injection into CSF.** Confocal microscope images showing QD (red) within the subcapsular sinus of a 140 μm thick section. The region delineated by the box in **(a)** can be seen in **(b)** at higher power. The capsule of the lymph node has been stained for collagen-IV (blue) and lymph node architecture is elucidated with Sytox green nuclear stain (green). Scale = 300 μm **(a)**, 150 μm **(b)**.

Confocal imaging of lymph node sections stained for collagen-IV showed QD signal within all submandibular lymph nodes beneath the collagen-IV-positive capsule 6 hours after CSF injection (Figure 4). Hematoxylin and eosin staining of the adjacent sections confirmed the presence of lymph node cytoarchitecture. QD signal in the superficial parotid and deep cervical lymph nodes of control mice was confirmed with histological analysis of neck sections.

## Discussion

Results from this study are in keeping with those from non-invasive imaging of CSF drainage to lymph nodes of the neck in larger species [7,8]. In rabbit [7,8] and cat [8], tracer injected into CSF of the lateral ventricle was non-invasively imaged draining to the cervical lymph nodes, however these studies did not specify the subset of cervical lymph nodes.

Compared to previous non-invasive approaches [8], a higher degree of sensitivity, with earlier signal detection was possible using our combined quantum dot and hyperspectral imaging approach. Although drainage of CSF to surgically exposed lymph nodes can be visualized as early as 2 min after tracer injection in mice [9], this is the first non-invasive imaging study to detect drainage as early as 20 min and at multiple time points after injection using the same animals.

In all 7 mice studied, CSF drained preferentially to the submandibular lymph nodes, though drainage to deep cervical lymph nodes was also noted in 2 mice. One study in rat showed India ink injected into CSF was found solely in deep cervical and lumbar lymph nodes [3], while another showed human serum albumin injected into CSF elicited an immune response in both deep and superficial cervical nodes [15]. In sheep, radioactive albumin drained preferentially to retropharyngeal and deep cervical lymph nodes [6]. It has been shown that lymphatics of the head and neck can drain either directly to deep cervical nodes, or indirectly via superficial nodes, such as the submandibular nodes [16]. The preferential location of QD in the submandibular lymph nodes may depend on tracer characteristics, anatomical differences between species, or imaging time points, and does not exclude the presence of alternate lymphatic drainage pathways. Future experiments using varying size and charge of tracers and multiple time points may be helpful to map the regional lymphatic drainage pathway(s).

Signal was not detected *in vivo* in the submandibular region of control mice, indicating that tracer leakage at the injection site was not responsible for signal observed in the submandibular region following CSF injection.

There are some potential shortcomings for these experiments. The tonicity of the injected QD solution prepared by the manufacturer did not match the tonicity of CSF and possible effects of this are unknown. The use of QDs with heavy metal core composition for *in vivo* studies has also been questioned due to the potential for toxicity. However, it has been demonstrated in animals that there is no appreciable toxicity even after breakdown of QDs *in vivo *[17]. Furthermore, the presence of signal in tissues below the threshold of detection for this imaging system cannot be excluded. Lastly, since this was a qualitative study, additional work should be undertaken to optimize this technique for quantitative imaging [18].

The CSF and brain interstitial fluid are highly interconnected, and lymphatic drainage plays an important immunological role here [19]. Non-invasive *in vivo* imaging techniques as described in this study may also be relevant to investigation of interstitial fluid outflow and CNS immunology.

## Conclusions

The combined use of QD nanoparticles and hyperspectral imaging may be suitable for longer-term studies due to its relatively non-invasive nature, and capacity for multiple longitudinal imaging sessions in the same mouse. It may present an advantage over more invasive methods that require prolonged deep anesthesia and immobilization that are known to slow lymphatic flow [20]. This *in vivo* approach to image lymphatic drainage provides a unique opportunity to further assess lymphatic drainage of CSF in mouse models of CNS disorders.

## Competing interests

The authors declare they have no competing interests.

## Authors’ contributions

EM completed all experimental components and wrote the paper. RLM and JA contributed to the design of experiments and to writing the paper. NG and YHY contributed to experimental design, interpretation of results, and to writing the paper. All authors read and approved the final manuscript.
